# De Novo RNA Sequencing and Transcriptome Analysis of *Sclerotium rolfsii* Gene Expression during Sclerotium Development

**DOI:** 10.3390/genes14122170

**Published:** 2023-12-02

**Authors:** Fanfan Wang, Xiaoyue Wang, Tao Tang, Yuanyuan Duan, Ting Mao, Xiaoliang Guo, Qingfang Wang, Jingmao You

**Affiliations:** 1 Key Laboratory of Biology and Cultivation of Chinese Herbal Medicines, Ministry of Agriculture and Rural Affairs, Institute of Chinese Herbal Medicines, Hubei Academy of Agricultural Sciences, Enshi 445000, China; 2 Hubei Engineering Research Center of Under-Forest Economy, Hubei Academy of Agricultural Sciences, Wuhan 430064, China; 3 Hubei Engineering Research Center of Good Agricultural Practices (GAP) Production for Chinese Herbal Medicines, Institute of Chinese Herbal Medicines, Hubei Academy of Agricultural Sciences, Enshi 445000, China

**Keywords:** *Sclerotium rolfsii*, transcriptome, sclerotium, weighted gene co-expression network analysis

## Abstract

*Sclerotium rolfsii* is a destructive soil-borne fungal pathogen that causes stem rot in cultivated plants. However, little is known about the genetic basis of sclerotium development. In this study, we conducted de novo sequencing of genes from three different stages of *S. rolfsii* (mycelia, early sclerotium formation, and late sclerotium formation) using Illumina HiSeq^TM^ 4000. We then determined differentially expressed genes (DEGs) across the three stages and annotated gene functions. STEM and weighted gene-co-expression network analysis were used to cluster DEGs with similar expression patterns. Our analysis yielded an average of 25,957,621 clean reads per sample (22,913,500–28,988,848). We identified 8929, 8453, and 3744 DEGs between sclerotium developmental stages 1 versus 2, 1 versus 3, and 2 versus 3, respectively. Additionally, four significantly altered gene expression profiles involved 220 genes related to sclerotium formation, and two modules were positively correlated with early and late sclerotium formation. These results were supported by the outcomes of qPCR and RNA-sequencing conducted on six genes. This is the first study to provide a gene expression map during sclerotial development in *S. rolfsii*, which can be used to reduce the re-infection ability of this pathogen and provide new insights into the scientific prevention and control of the disease. This study also provides a useful resource for further research on the genomics of *S. rolfsii*.

## 1. Introduction

*Sclerotium rolfsii* Sacc. is a major soil-borne pathogen with a worldwide distribution that infects over 500 species of vegetables, grains, Chinese herbal medicines, and ornamental crops in warm-temperate, subtropical, and tropical regions [1]. This pathogen is the source of multiple diseases, including seedling damping-off, crown and root rot, and dry rot canker [2], which are commonly referred to as southern blight. The recent influence of climate warming, high-density planting, successive cropping obstacles, and other factors have led to a continued increase in southern blight in various crop-producing areas, with a resulting yield loss of 10–80% and serious economic losses [3]. Moreover, new hosts such as mung bean, *Bletilla orchid*, and *Artocarpus heterophyllus* continue to be detected [4,5,6]. Southern blight is especially difficult to control because *S. rolfsii* produces melanin-containing, thick-walled, resistant bodies that survive in the soil for over four years [7].

*S. rolfsii* predominantly overwinters in the soil as sclerotium and as mycelium on diseased plant residues. High humidity and warm conditions in the following year allow the sclerotium to germinate and grow mycelium, which spreads through cracks in the soil to neighboring plants [2]. The mycelium directly invades the host from the wound or the epidermis of the plant root or stem, causing plant disease and rot. Mature sclerotium on the infected strain can then be spread via rain, insects, and farm operations, causing re-infection [8]. Sclerotium is the core structure of the life history and infection of *S. rolfsii*; therefore, clarifying its developmental mechanism will not only elucidate the developmental biology of sclerotium but also provide a theoretical and molecular basis for the further analysis of its pathogenic mechanism.

Sclerotia are the asexual structures formed from the aggregation of fungal mycelia. The biogenesis of sclerotia is related to lipid peroxidation and induced by oxidative stress as a pathogen consumes carbon sources [9]. Xing et al. [10] reported that an increase in oxalic acid levels inhibits sclerotial initiation at the collar region of the host. In addition, sclerotium exudates directly affect the development and maturation of sclerotium [11]. Scholars have also studied the effects of air, light, and soil on the size, quantity, and germination rate of sclerotium [12]. Numerous environmental factors induce sclerotial development, including cold, drought, low nutrition, oxidative stress, hyphal damage, and pH imbalance [13,14]. However, the genes that control these processes and that are differentially expressed between the filamentous mycelium and sclerotia of *S. rolfsii* remain unknown.

Next-generation sequencing technologies such as Illumina sequencing are now commonly applied in comprehensive transcriptional studies. Illumina sequencing, which is cost-effective and has a higher output than traditional methods, yields highly accurate measurements of gene expression during transcriptome analysis because the sequencing reads can be counted and mapped to a genome or annotated transcripts [15,16,17], facilitating the genome-wide identification of coding sequences, gene structures, and alternative splicing. The transcriptomes of several fungi have already been sequenced (e.g., *Aspergillus flavus* [18], *Rhizoctonia solani* [19], and *Wolfiporia cocos* [20]); however, these data are not available for *S. rolfsii*, and very few studies have investigated sclerotia formation in *S. rolfsii* [21].

Therefore, to reveal the molecular mechanisms of sclerotial development in *S. rolfsii*, we used Illumina HiSeq^TM^ 4000 for transcriptome analysis at three stages of sclerotium development along with RNA sequencing and de novo assembly. The results reveal the developmental-stage-specific gene expression patterns of sclerotia, as well as multiple differentially expressed genes associated with the development process of *S. rolfsii*. Moreover, bioinformatic analysis was used to analyze the gene expression profiles and related gene modules exhibiting significant changes during sclerotial development. These genes reveal the major metabolic processes and signaling pathways during sclerotial hardening in *S. rolfsii*. This study provides valuable insights into the regulatory mechanisms that control sclerotial development, which can be used to design new control strategies for southern blight.

## 2. Materials and Methods

### 2.1. Strains and Specimen Collection

**Figure 1:**
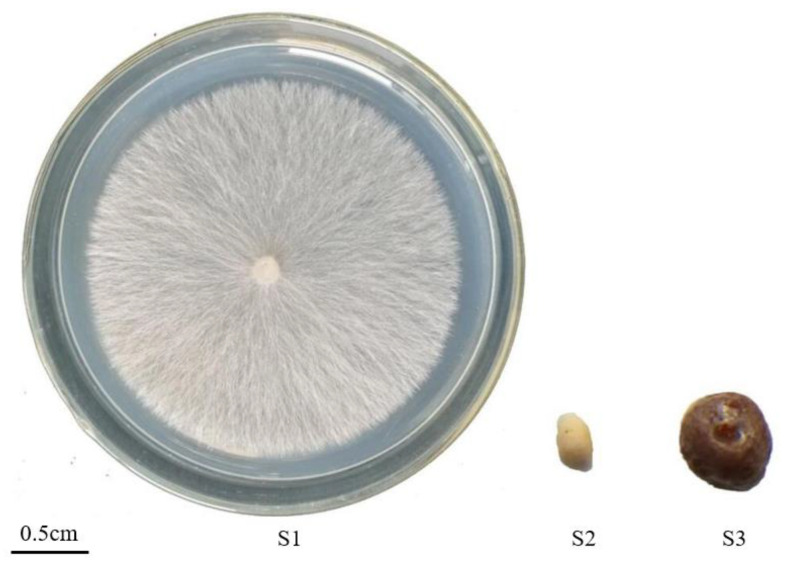
Different developmental stages of *Sclerotium rolfsii*.

*S. rolfsii* strain CB1 was obtained from the *Macleaya cordata* nursery at Hunan Agricultural University, Changsha, China [22]. Mycelia were grown on a cellophane membrane placed on potato dextrose agar medium at 28 °C for three days (stage 1, S1) before collection. Sclerotia were also collected after 10 (stage 2, S2) and 30 days (stage 3, S3) (Figure 1). Samples were then immediately frozen in liquid nitrogen for total RNA extraction. Each stage had three biological replicates.

### 2.2. RNA Extraction

Total RNA from S1, S2, and S3 was extracted using a TRIzol reagent kit (Life Technologies Inc., Carlsbad, CA, USA). Degradation and contamination of RNA were verified using 1% RNase-free agarose gel electrophoresis, and the purity and integrity were determined using a Nanodrop 2000 (Thermo Fisher Scientific, Waltham, MA, USA) and 2100 Bioanalyzer (Agilent Technologies, Santa Clara, CA, USA). High-quality RNA (RNA Integrity Number [RIN] scores > 7.5) was used for subsequent experiments, with three biological replicates for each stage.

### 2.3. Library Construction and Sequencing

Sample mRNA was enriched using oligo (dT) beads, treated with fragmentation buffer, and reverse-transcribed into cDNA using random primers. Second-strand cDNA was synthesized using DNA polymerase I, RNase H, dNTP, and a buffer. Next, cDNA was purified using 1.8× Agencourt AMPure XP Beads, end-repaired, affixed with poly(A) tails, and ligated to Illumina sequencing adapters. Ligation products were size-selected using agarose gel electrophoresis, PCR-amplified, and sequenced in an Illumina HiSeq^TM^ 4000 by Gene Denovo Biotechnology Co. (Guangzhou, China).

### 2.4. De Novo Assembly

The transcriptome was assembled in the modular program Trinity [23], which includes three components: Inchworm, Chrysalis, and Butterfly. Inchworm assembles reads using a greedy k-mer-based approach, resulting in a collection of linear contigs. Chrysalis then clusters related contigs that correspond to sections of alternatively spliced transcripts or otherwise unique regions of paralogous genes and subsequently builds de Bruijn graphs for each cluster. Finally, Butterfly analyzes the paths taken by reads and read pairings in the corresponding de Bruijn graph and then outputs one linear sequence for each alternatively spliced isoform and transcript derived from paralogous genes.

All raw sequencing data were submitted to the Genome Sequence Archive (GSA, https://ngdc.cncb.ac.cn/gsub/ (accessed on 30 January 2023)) under the BioProject accession number CRA009668.

### 2.5. Unigene Functional Analysis

Unigenes were annotated using BLASTx (http://www.ncbi.nlm.nih.gov/BLAST/ (accessed on 17 March 2022)) with a threshold of E-value = 10^−5^. The following databases were used for annotation: Non-redundant Protein (Nr) (http://www.ncbi.nlm.nih.gov/ (accessed on 29 June 2010)), Swiss-Prot (http://www.expasy.ch/sprot/ (accessed on October 2020)), Kyoto Encyclopedia of Genes and Genomes (KEGG) (http://www.genome.jp/kegg/ (accessed on 1 November 2022)), clusters of orthologous groups (KOG/COG) (https://www.ncbi.nlm.nih.gov/research/cog/ (accessed on March 2022)), and Gene Ontology (GO) (https://www.geneontology.org/ (accessed on December 2022)). Annotations were determined based on the best alignment.

### 2.6. Gene Quantification

Gene abundance was calculated and normalized to reads per kilobase per million reads (RPKM) [24] using the following formula: RPKM = (106C)/(NL/103); here, C is the number of reads uniquely mapped to a given unigene, N is the total number of reads mapped to all unigenes, and L is the length in bases of the unigene. The reads of each unigene belonging to the same pathway were summed. For selected pathways, the number of reads per stage was transformed into Z-scores, clustered, and plotted in the R package Heatmap 4.2 (http://www.r-project.org/ (accessed on 13 March 2023)).

### 2.7. Analysis of Differentially Expressed Genes

The R package “edge” was used to identify differentially expressed genes (DEGs) based on fold change (FC) ≥ 2 and false discovery rate < 0.05. Significant DEGs were then subjected to GO and KEGG enrichment analyses. Significantly enriched terms were those with a corrected *p* < 0.05. Next, principal component analysis was performed on the transcriptomes of samples across the three sclerotial developmental stages (S1, 2, and 3) using the R package “psych” with standard settings, and a heatmap was generated in the “NMF” package. Different samples were compared using the COR function in the “stats” R package; Pearson’s correlations were reported only for the accuracy of observations. After normalizing (log^2^ transformation) expression data, DEGs (|log_2_FC| > 1) were clustered using STEM [25] to obtain expression profiles related to sclerotium formation. The threshold cluster number and correlation coefficient were set to 20 and 0.7, respectively. Significance was set at *p* < 0.05.

### 2.8. Weighted Gene Co-Expression Network Analysis

Genes with standardized RPKM ≥ 1 were used for co-expression analysis. Co-expression modules were constructed using the weighted gene co-expression network analysis version 1.47 package in R version 4.2 [26], and genes with similar expression patterns were assigned to the same module. Biological modules were classified based on gene expression levels and module eigengene (ME) similarity. Parameters for module classification were set as follows: minimum power = 5, ME = 0.65, and minModuleSize = 50. Pearson correlation coefficients (r) were calculated between MEs. Modules were associated with sclerotium formation if r > 0.5 and *p* < 0.05. Gene function was then further explored using GO and KEGG analyses. Lastly, intramodular connectivity between genes and intermodular correlations was calculated via weighted gene co-expression network analysis.

### 2.9. Quantitative Real-Time PCR (qPCR) Validation of mRNA Expression

To verify the sequencing accuracy, 10 DEGs were randomly selected for qPCR, with α-tubulin serving as the reference gene. Specific primers (Table S1) were designed using Primer 5 based on sequenced transcripts. The thermocycling profile was as follows: 95 °C for 2 min, followed by 40 cycles at 95 °C for 10 s and 60 °C for 30 s. Relative expression was calculated using 2^−ΔΔCt^. Values for each biological replicate were calculated using three technical replicates.

## 3. Results

### 3.1. RNA-Seq of S. rolfsii during Sclerotial Development

Illumina sequencing generated 27,778,288, 27,374,584, and 23,420,774 raw reads in the three independent biological replicates of S1; 25,401,562, 23,896,106, and 27,575,880 raw reads in S2; and 27,697,540, 29,499,244, and 25,507,578 raw reads in S3 (Table 1). Removing excess adaptors and low-quality reads from the nine libraries yielded 233,618,590 reads, with a Q20 percentage of 98.64% and a Q30 percentage of 95.66%. We obtained an average of 25,957,621 reads per sample (range = 22,913,500–28,988,848) (Table 1). All the clean reads, with 36,165,475 assembled bases, were clustered into 28,158 unigenes, averaging 1284 bp (range = 201–16,402 bp). The GC percentage was 48.29%, and N50 was 2379 (Table 2).

### 3.2. Functional Annotation

The BLASTX results demonstrated that 14,763, 9188, 10,275, and 3096 unigenes significantly matched with proteins from the Nr, SwissProt, GO, and KEGG databases, respectively (Table 3). However, 12,836 unigenes were absent from the tested databases, likely because they are noncoding or new genes with unknown functions. According to the species distribution against Nr, 1142 *S. rolfsii* unigenes matched those from *Hypsizygus marmoreus*. The next species with the most unigenes matched with *S. rolfsii* was *Moniliophthora roreri* (838 genes), followed by *Fibulorhizoctonia* sp. (763), *Lentinula edodes* (667), *Rhizopogon vinicolor* (473), *Neolentinus lepideus* (446), *Stereum hirsutum* (433), *Gloeophyllum trabeum* (415), *Grifola frondosa* (393), and *Plicaturopsis crispa* (391) (Table S2).

### 3.3. Classification of GO and KOG

**Figure 2:**
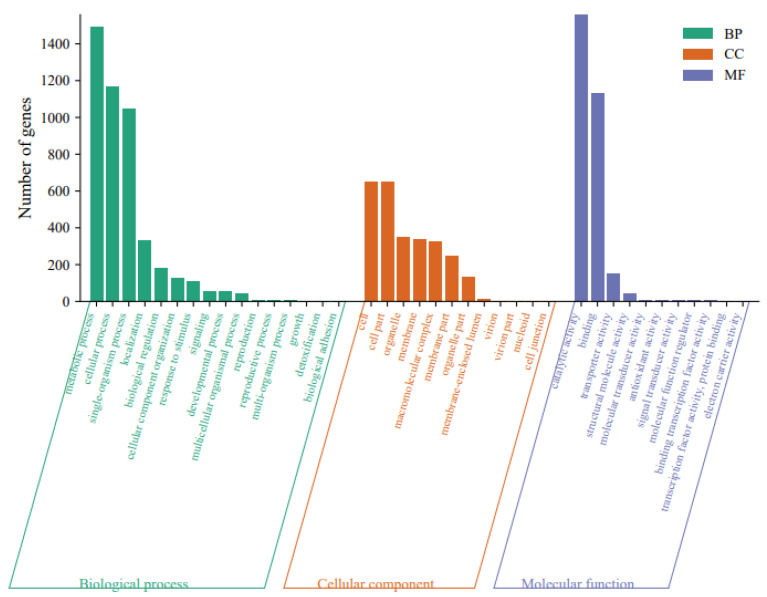
GO enrichment analysis of the *Sclerotium rolfsii* transcriptome. Results are grouped into biological process, cellular component, and molecular function. *Y*-axis represents the number of genes in a category.

**Figure 3:**
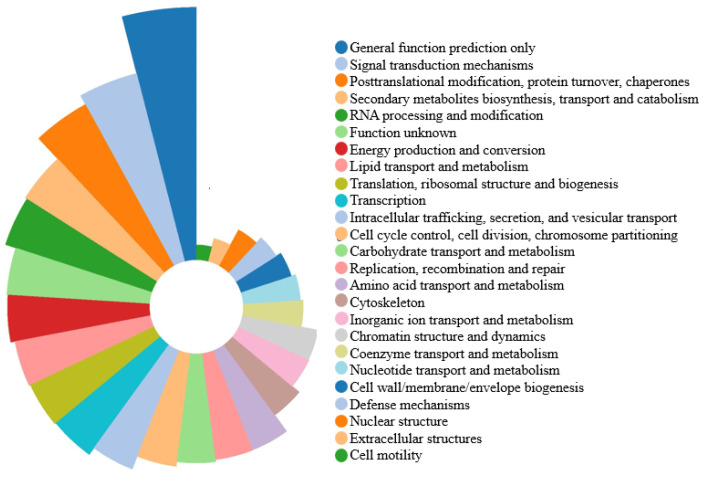
KOG enrichment analysis of the *Sclerotium rolfsii* transcriptome. The unigenes were categorized into 25 sub-categories.

The results of GO analysis classified 10,275 annotated unigenes largely into biological processes, followed by molecular functions and cellular components (Table S3). Within biological processes, “metabolic process”, “cellular process”, and “single-organism process” were the dominant enriched terms. Within molecular functions, the dominant enriched terms were “catalytic activity” and “binding.” Within cellular components, “cell” and “cell part” were enriched (Figure 2). Overall, “catalytic activity”, “metabolic process”, and “cellular process” were key enriched terms, reflecting the importance of metabolism during *S. rolfsii* sclerotial development. The KOG analysis classified 17,094 unigenes into 25 categories (Figure 3 and Table S4), with the largest being “general function prediction only” (2882, 16.86%), followed by “signal transduction mechanisms” (1699, 9.94%), “posttranslational modification, protein turnover, chaperones” (1528, 8.94%), “secondary metabolites biosynthesis, transport and catabolism” (1100, 6.44%), and “RNA processing and modification” (1074, 6.28%).

### 3.4. KEGG Pathway Analysis

The KEGG analysis assigned 3096 annotated unigenes to 112 pathways. “Metabolic pathways” contained the majority of *S. rolfsii* unigenes, mainly involving “carbon metabolism” (6.07%), “starch and sucrose metabolism” (4.55%), “amino sugar and nucleotide sugar metabolism” (3.81%), “purine metabolism” (3.71%), “oxidative phosphorylation” (3.46%), “arginine and proline metabolism” (2.84%), “glycine, serine and threonine metabolism” (2.78%), and “tryptophan metabolism” (2.71%) (Table S5). In addition, pathways involving “biosynthesis of amino acids” (6.88%), “RNA transport” (5.56%), and “protein processing in endoplasmic reticulum” (4.04%) comprise a large proportion of *S. rolfsii* unigenes.

### 3.5. Functional Distribution of DEGs between Mycelium and Sclerotial Developmental Stages

**Figure 4:**
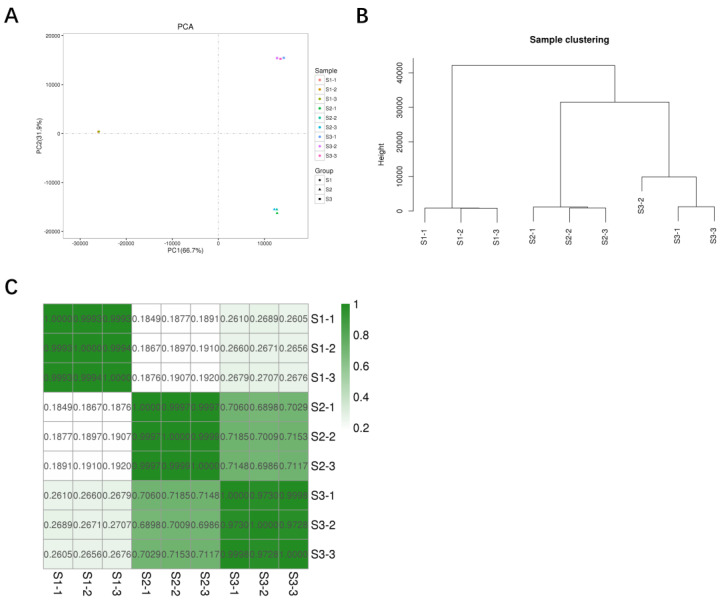
Principal component analysis (**A**), cluster analysis (**B**), and correlation analysis (**C**) results of DEGs at different sclerotial developmental stages.

The principal component analysis demonstrated that all three biological replicates exhibited similar expression patterns, indicating good repeatability (Figure 4A). Additionally, clustering analysis revealed that S2 and S3 formed a group together, separate from S1 (Figure 4B). Subsequent Pearson correlations and heat maps supported the clustering results, indicating that the S2 and S3 samples had similar functional distributions of DEGs, which differed from the distribution of the S1 samples (Figure 4C and Figure S1).

**Figure 5:**
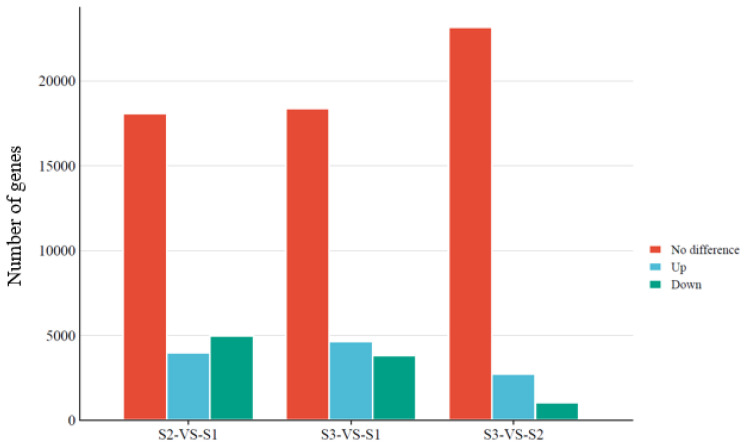
Comparison of gene expression profiles between S1, S2, and S3 samples.

We then compared the gene expression profiles among S1, S2, and S3. Compared with S1, 3975, 4954, and 18,073 genes were upregulated, downregulated, and not differentially expressed in S2, respectively (Figure 5 and Table S6), while 4651, 3820, and 18,387 genes were upregulated, downregulated, and not differentially expressed in S3 (Figure 5 and Table S7). Additionally, 2716, 1028, and 23,161 genes were upregulated, downregulated, and not differentially expressed in S3 versus S2 (Figure 5 and Table S8).

Next, we performed functional analyses of DEGs in the S2-vs-S1, S3-vs-S1, and S3-vs-S2 groups. In S2-vs-S1, DEGs were enriched in thirty functional categories, with the top five being metabolic processes (620 genes), catalytic activity (615 genes), cellular processes (429 genes), binding (415 genes), and single-organism processes (400 genes) (Table S9). In S3-vs-S1, the DEGs were categorized into twenty-six functional groups, with the same top five, albeit ordered differently: catalytic activity (633 genes), metabolic processes (622 genes), cellular processes (445 genes), binding (413 genes), and single-organism processes (403 genes) (Table S10). Finally, in the S3-vs-S2 group, the DEGs were categorized into 21 functional groups, with the top five being catalytic activity (196 genes), metabolic processes (149 genes), single-organism processes (132 genes), cellular processes (125 genes), and binding (120 genes) (Table S11).

**Figure 6:**
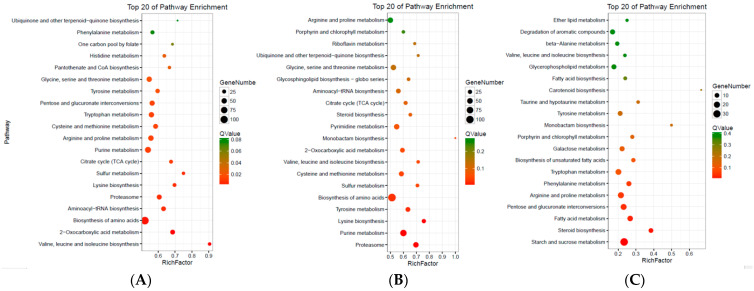
The KEGG enrichment analysis of DEG in S2-vs-S1 (**A**), S3-vs-S1 (**B**), and S3-vs-S2 (**C**).

Lastly, KEGG analysis demonstrated that amino acid biosynthesis was the top enriched metabolic pathway in both S2-vs-S1 and S3-Vvs-S1 (Figure 6 and Tables S12 and S13). In contrast, the top enriched pathway in S3-vs-S2 was starch and sucrose metabolism (Figure 6 and Table S14), indicating similarly enriched pathways in the S2 and S3 samples.

### 3.6. STEM Analysis of DEGs Involved in Sclerotium Formation

**Figure 7:**
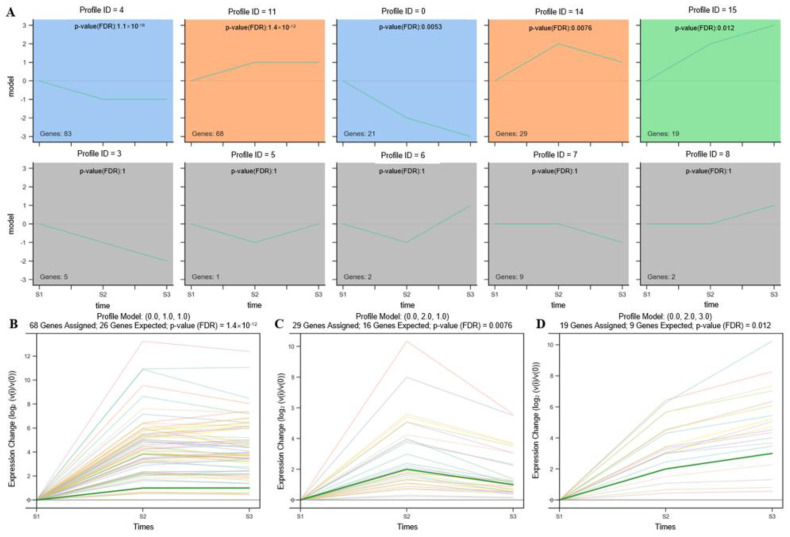
STEM analysis of all DEGs based on sclerotium formation time series data. (**A**) Ten expression profiles; (**B**) expression trends for genes in profile 11; (**C**) expression trends for genes in profile 14; (**D**) expression trends for genes in profile 15. Each line in the figure (**B**–**D**) represents one gene.

STEM analysis uncovered five significant gene-expression profiles (profiles 4, 11, 0, 14, and 15; *p* < 0.05) based on 220 DEGs related to sclerotium formation. Profiles 0 and 15 exhibited clear gene expression trends, whereas profiles 4, 11, and 14 did not (Figure 7A). The 21 DEGs in profile 0 were significantly downregulated (*p* = 0.0053, Figure 7A) throughout sclerotium development, whereas the 19 DEGs in profile 15 were significantly upregulated (*p* = 0.012, Figure 7D). Gene expression in profile 14 (*p* = 0.0076, 29 DEGs) was upregulated during early sclerotium development and then downregulated during late sclerotium development, with an overall trend of upregulation (Figure 7C). Gene expression in profile 11 (*p* = 1.4 × 10^−12^, 68 DEGs) was upregulated during early sclerotium development, whereas that in profile 4 (*p* = 1.1 × 10^−16^, 83 DEGs) was downregulated during the same period (Figure 7B).

### 3.7. Correlation between Modules and Identification of Key Modules

**Figure 8:**
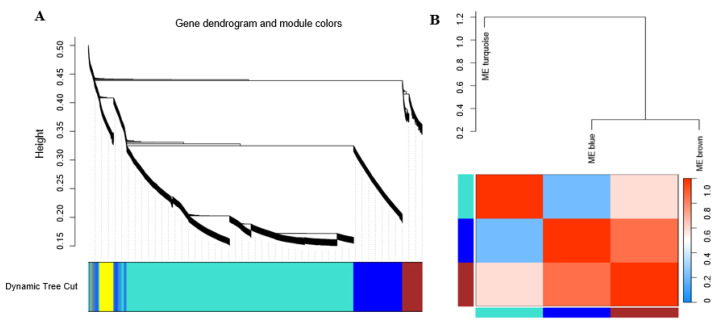
(**A**) The cluster dendrogram of gene in *Sclerotium rolfsii*. Each branch in the figure represents one gene, and every color below represents one co-expression module. (**B**) Eigengene dendrogram and eigengene adjacency heatmap. Every color below represents one co-expression module.

We generated three final modules after merging similar modules (Figure S2), the first of which contained 5377 genes, the second contained 1286 genes, and the third contained 796 genes. The results of the interaction analysis and the heatmap revealed that the modules were independent, demonstrating unrelated gene expression within each module (Figure 8A). We also identified and clustered eigengenes to explore co-expression across all modules. This analysis revealed three modules divided into two clusters, a result that was consistent with the eigengene-based heatmap (Figure 8). Furthermore, the MEs of the second and third modules were both highly correlated with sclerotium formation (Figure 8B), specifically the late and early stages, respectively. In contrast, the first module was positively correlated with mycelium formation.

### 3.8. Validation of Differential Expression Profiles Using qPCR

**Figure 9:**
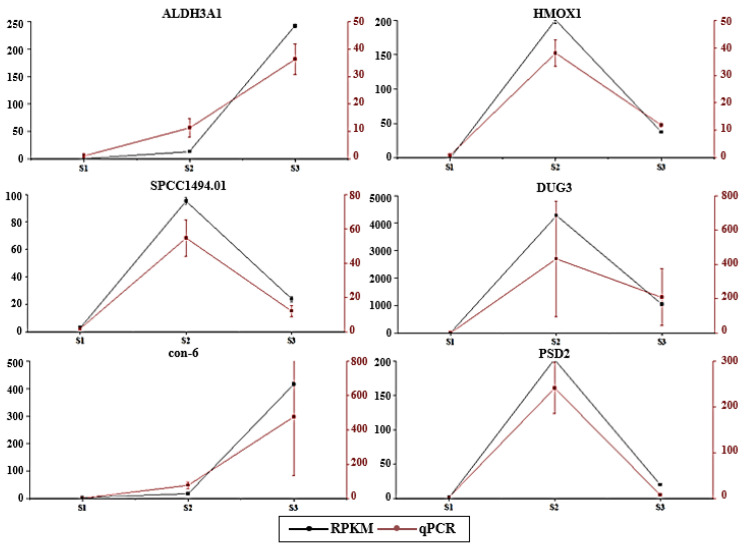
qPCR analysis of six genes used for the validation of RNA-Seq data.

The qPCR results validated the RNA-Seq data for two upregulated (ALDH3A1 and con-6) and four downregulated (HMOX1, SPCC1494.01, DUG3, and PSD2) genes. The results showed consistent differences between qPCR and RNA-Seq (Figure 9). The observed similarity between the qPCR and RNA-Seq data supports the validity of our results.

## 4. Discussion

In this study, we successfully identified the potential genetic mechanisms underlying the pathology of *S. rolfsii*, which causes southern blight disease in numerous crops. Previously, Song et al. [27] cultured *S. rolfsii* in sucrose and glucose media to investigate global metabolic and genetic changes using LC-MS combined with RNA-Seq. Furthermore, Schmid applied massively parallel short-read 454 pyrosequencing to identify DEGs under scleroglucan-producing and non-producing conditions [21]. However, our study is the first to provide de novo comparative transcriptome data related to sclerotial development in this pathogenic species, analyzing transcriptomic variation across the mycelium, early sclerotium, and mature sclerotium stages. Transcriptome sequencing has previously been widely applied to investigate the morphological development of pathogenic bacteria [15]. For example, Yu et al. [28] used transcriptomics to compare gene expression differences in the fruit body development and sporulation stages of *Ustilaginoidea virens*, thereby clarifying sexual reproduction mechanisms.

Here, we identified key genes in sclerotium biosynthesis through the Illumina sequencing of the *S. rolfsii* transcriptome. We successfully annotated 15,322 (54.41%) unigenes and performed functional analyses (GO, KEGG, and KOG). Furthermore, we performed DEG analysis across the three stages of sclerotial development and identified thousands of upregulated and downregulated genes between S1-vs-S2, S1-vs-S3, and S2-vs-S3. Overall, this study provides a novel perspective based on transcriptome sequences of developmental stages and annotated gene functions, which can aid further research on sclerotium formation in *S. rolfsii*. Functional analysis indicated that amino acid biosynthesis was the most enriched metabolic pathway between S2-vs-S1 and S3-vs-S1, and this result is consistent with the significant enrichment of multiple amino acid pathways during sclerotium formation in Botrytis cinerea [29]. Hence, amino acid metabolism appears to play an important role in sclerotia formation. S3 is similar to S2 in that starch and sucrose metabolism are the most enriched pathways. Wu et al. [20] demonstrated that fewer transcriptional changes occurred during earlier developmental stages and that molecular mechanisms became increasingly sophisticated with greater sclerotium maturity. Therefore, future studies should further examine DEGs at different sclerotium developmental stages to elucidate the biological mechanisms involved.

Numerous studies have shown that reactive oxygen species are necessary for sclerotium initiation and are accompanied by increased lipid peroxidation levels [30]. As the superoxidized state is harmful to cells, the internal defense mechanisms of organisms have evolved to produce antioxidant molecules such as glutathione, superoxide dismutase, catalase, and glutathione peroxidase, which neutralize the accumulation of reactive oxygen species [31]. The expression level of NADPH-related unigenes in *S. rolfsii* was higher in S2 and S3; this finding is consistent with the results reported by Schurmann et al. [32] on the sclerotial formation of *Botrytis cinerea*. In addition, unigenes involved in autophagy were consistently highly expressed in S2 and S3. Autophagy is a highly conserved cellular degradation process in eukaryotes that is typically maintained at very low levels [33]. However, autophagy is induced under stress conditions and can reduce reactive oxygen species levels, thereby accelerating protein elimination [34]. Moreover, other stress-regulatory proteins, such as heat shock protein and redox enzyme FAD/NAD-binding protein, were also highly expressed in S2. To protect an organism from harmful reactive oxygen species and regulate the balance between cell survival and death, heat shock proteins may be produced [35]. This study shows that *S. rolfsii* responds to oxidative stress through different pathways during sclerotium development.

Signal transduction pathways are essential in sclerotium formation. The cAMP signaling pathway mediated by G protein-coupled receptors is an important signal transduction pathway. It plays an important role in fungal sclerotial formation [36]. In this study, the expression of G protein-coupled receptor family A increased at S2, indicating a close relationship between cAMP and sclerotium formation in *S. rolfsii*. Kinases and phosphokinases influence sclerotium formation by controlling phosphorylation and dephosphorylation in signaling pathways. The expression of kinase-like proteins in *S. rolfsii* increases in S2, which is consistent with the results of Harel et al. [37]. In addition, carbonic anhydrase, carbohydrate-binding module, and oxalate decarboxylase showed a continuous increasing trend throughout the three stages. Carbonic anhydrase superfamilies are prevalent in all organisms and are essential for fungal pathogen perception and controlling sexual development [38]. Recent findings also highlight the many functions of CBM32, suggesting its direct involvement in the pathogenesis of *S. rolfsii* [39]. In summary, the results showed that sclerotial development is regulated by many factors and involves complex signal transduction pathways, which should be further explored to provide a new target for inhibiting sclerotial development.

Our study confirms the gene expression patterns and stage-specific transcriptomic changes in sclerotial development. Furthermore, unigenes, which are differentially regulated during sclerotia formation, are involved in the biosynthesis of secondary metabolites, autophagy, and active oxygen metabolism. The annotated unigenes in this study will provide useful information for disease control strategies designed to prevent sclerotium formation.

## 5. Conclusions

To the best of our knowledge, this is the first study to sequence the transcriptome of *S. rolfsii* at three different developmental time points. By identifying the key genes in sclerotium biosynthesis, this study elucidates the expression patterns at different stages of sclerotium maturation. Furthermore, the functional annotation of these genes offered insights into the molecular mechanisms underlying sclerotium development in *S. rolfsii*. In conclusion, our study represents an important contribution to *S. rolfsii* pest management efforts by revealing the target genes that may reduce pathogenicity.

## Figures and Tables

**Table 1:** Summary of transcriptome assembly after filtering.

Annotation Database	No. of Annotation	Percent of Annotation (%)
Total Unigenes	28,158	100%
NR	14,763	52.43%
SwissProt	9188	32.63%
GO	10,275	36.49%
KEGG	3096	11%
Annotation genes	15,322	54.41%
Without annotation gene	12,836	45.59%

**Table 2:** Summary of de novo assembly of unigenes.

Sample Name	Raw Reads	Clean Reads	Clean Bases	Q20 (%)	Q30 (%)	Content (%)
S1-1	27,778,288	27,173,090	2.72G	98.56	95.5	52.17
S1-2	27,374,584	26,896,318	2.69G	98.7	95.8	52.25
S1-3	23,420,774	22,913,500	2.29G	98.58	95.5	52.3
S2-1	25,401,562	24,936,756	2.49G	98.69	95.83	51.62
S2-2	23,896,106	23,481,220	2.35G	98.69	95.77	51.59
S2-3	27,575,880	27,038,696	2.70G	98.61	95.59	51.67
S3-1	27,697,540	27,164,438	2.72G	98.6	95.55	52.14
S3-2	29,499,244	28,988,848	2.90G	98.69	95.82	51.74
S3-3	25,507,578	25,025,724	2.50G	98.62	95.57	52.21
Total	238,151,556	233,618,590	23.36	887.74	860.93	467.69

**Table 3:** All-in-one list of annotations.

Genes Num	GC Percentage	N50	Max Length	Min Length	Average Length	Total Assembled Bases
28,158	48.29%	2379	16,402	201	1284	36,165,475

## Data Availability

All raw sequencing data are openly available in Genome Sequence Archive (GSA, https://ngdc.cncb.ac.cn/gsub/ (accessed on 30 January 2023)) of BioProject accession number CRA009668.

## Supplementary Materials

The following supporting information can be downloaded at https://www.mdpi.com/article/10.3390/genes14122170/s1. Table S1: Specific primers for 10 verified genes used for qPCR. Table S2: Species distribution against Nr. Table S3: GO classification. Table S4: KOG classification. Table S5: Pathway list of genes. Table S6: Gene expression profiles in S1-vs-S2. Table S7: Gene expression profiles in S1-vs-S3. Table S8: Gene expression profiles in S2-vs-S3. Table S9: Functional analyses of DEG in S1-vs-S2. Table S10: Functional analyses of DEG in S1-vs-S3. Table S11: Functional analyses of DEG in S2-vs-S3. Table S12: Pathway in S1-vs-S2. Table S13: Pathway in S1-vs-S3. Table S14: Pathway in S2-vs-S3. Figure S1: Significant differential gene heatmap. Figure S2: Cluster dendrogram of (A) module eigengenes and (B) genes.
