# Supplementary material for: De Novo RNA Sequencing and Transcriptome Analysis of Sclerotium rolfsii Gene Expression during Sclerotium Development
Source: Genes (Basel). 2023 Dec 2;14(12):2170. doi: 10.3390/genes14122170 (PMC10743028; doi:10.3390/genes14122170)
Supplement: Supplementary file 1 [file genes-14-02170-s001.zip › Figure S2.pdf]

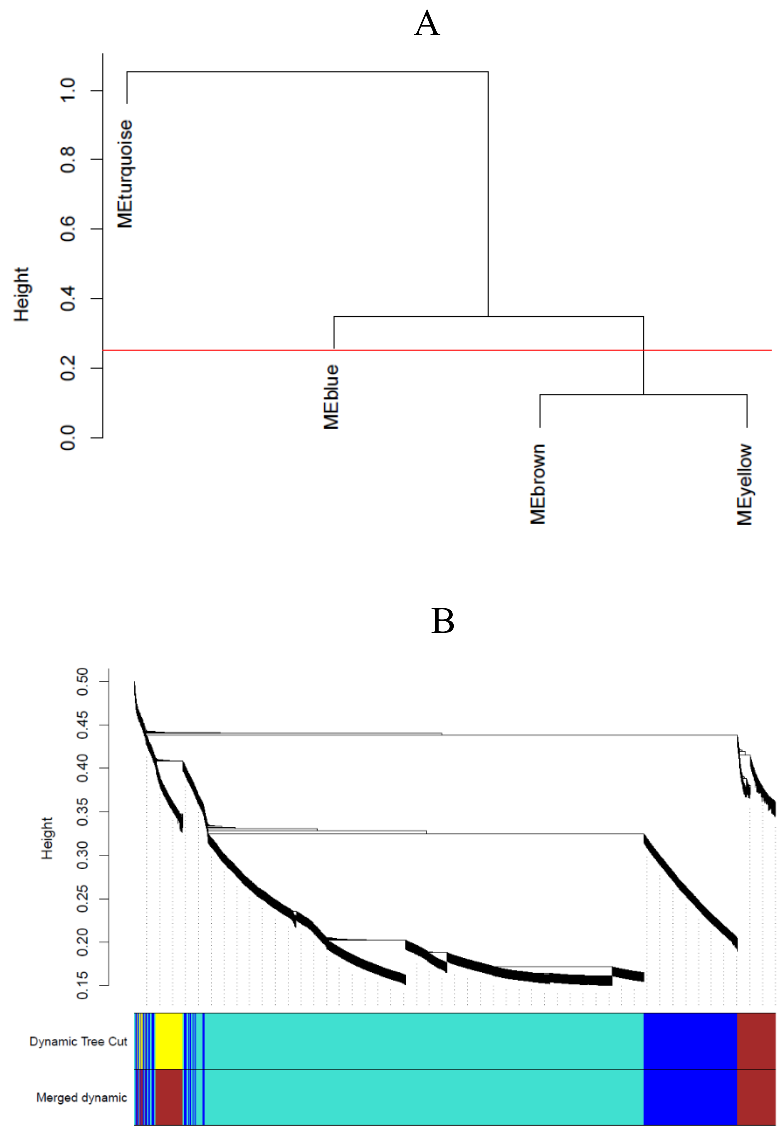

Figure S2 (A) The cluster dendrogram of module eigengenes. (B) The cluster dendrogram of genes . Each branch in the figure represents one gene, and every color below represents one co-expression module.
