# Supplementary figures and images for: De Novo RNA Sequencing and Transcriptome Analysis of Sclerotium rolfsii Gene Expression during Sclerotium Development
Source: Genes (Basel). 2023 Dec 2;14(12):2170. doi: 10.3390/genes14122170 (PMC10743028; doi:10.3390/genes14122170)

Significant Differential Gene Heatmap

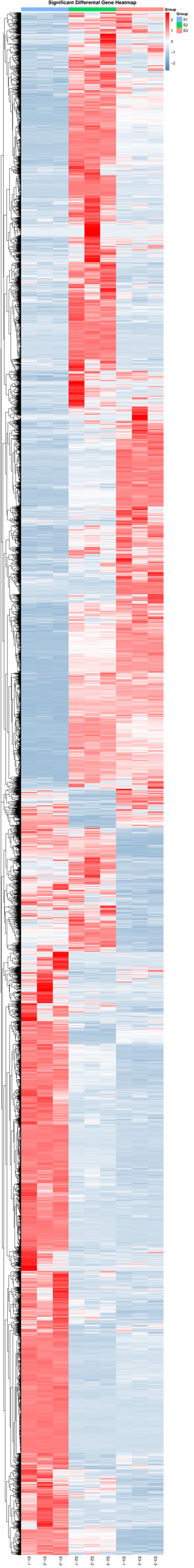

Supplement: Supplementary file 1 [file genes-14-02170-s001.zip › Figure S1.pdf]
